# Multifactor-dimensionality reduction versus family-based association tests in detecting susceptibility loci in discordant sib-pair studies

**DOI:** 10.1186/1471-2156-6-S1-S146

**Published:** 2005-12-30

**Authors:** Yan Meng, Qianli Ma, Yi Yu, John Farrell, Lindsay A Farrer, Marsha A Wilcox

**Affiliations:** 1 Genetics Program, Department of Medicine, School of Medicine, Boston University, Boston, MA, USA; 2 Bioinformatics Program, Boston University, Boston, MA, USA

## Abstract

Complex diseases are generally thought to be under the influence of multiple, and possibly interacting, genes. Many association methods have been developed to identify susceptibility genes assuming a single-gene disease model, referred to as single-locus methods. Multilocus methods consider joint effects of multiple genes and environmental factors. One commonly used method for family-based association analysis is implemented in FBAT. The multifactor-dimensionality reduction method (MDR) is a multilocus method, which identifies multiple genetic loci associated with the occurrence of complex disease. Many studies of late onset complex diseases employ a discordant sib pairs design. We compared the FBAT and MDR in their ability to detect susceptibility loci using a discordant sib-pair dataset generated from the simulated data made available to participants in the Genetic Analysis Workshop 14. Using FBAT, we were able to identify the effect of one susceptibility locus. However, the finding was not statistically significant. We were not able to detect any of the interactions using this method. This is probably because the FBAT test is designed to find loci with major effects, not interactions. Using MDR, the best result we obtained identified two interactions. However, neither of these reached a level of statistical significance. This is mainly due to the heterogeneity of the disease trait and noise in the data.

## Background

It is commonly believed that complex diseases are caused not by single genes acting alone, but by multiple genes interacting with one another. Due to the large number of single-nucleotide polymorphisms (SNPs) available in a genome-wide scan, the computational burden of testing each locus for main effects and all possible pair-wise, 3-way, and even higher-order interactions is overwhelming. One approach is to first identify a smaller number of candidate SNPs, using linkage analysis or a candidate gene approach. With a refined list, a more thorough statistical analysis can be performed. At this second stage, a univariate test is commonly used, which we refer to as single-locus method. Family-based association tests (FBAT) are used for pedigree data [1] and a chi-square test for case-control data. When SNPs have large interaction effects, but very small marginal effects in the population, the single-locus method will result in low power for detecting them.

There are several multilocus approaches that consider interactions of multiple genes and environmental factors in identifying susceptibility loci for complex diseases [2-5]. The multifactor-dimensionality reduction (MDR) method [2] was developed specifically to detect higher-order interactions among polymorphisms even when the marginal effects are very small. This method assumes a dichotomous trait. MDR is an extension of a combinatorial partitioning method [3]. It reduces the dimensionality of multilocus information to improve the identification of polymorphism combinations associated with disease risk. Currently, MDR is applicable only for case-control and discordant sib pair study designs. Investigators have used MDR successfully in the identification of gene × gene interactions in data from case-control studies of sporadic breast cancer [2] and essential hypertension [6]. Previous empirical studies have demonstrated that this method can take advantage of the information available in case-control studies and thereby maximize the statistical power in a given sample. This has been shown in the identification of higher-order interactions in simulated data [2,7]. However, these tests are based on case-control data, not family-based discordant sib pairs. Many studies of late onset complex diseases, such as Alzheimer disease, use discordant sib-pair designs because when the patients are diagnosed as affected in their 70s, their parents are usually not alive. Our goal was to compare the ability of FBAT and MDR to detect multiple susceptibility loci in family-based discordant sib-pair data.

## Methods

We chose to use to the simulated data provided to participants in the Genetic Analysis Workshop 14 (GAW14). To avoid analysis bias, we did the analysis without knowing the real answers prior to the GAW14 conference. Because we used simulated data with a fictitious trait, there were no a priori candidate genes to consider, so we used a positional approach to identify candidate regions. First, we performed linkage analysis using microsatellite markers with GENEHUNTER-PLUS. Next, we identified candidate regions near linkage peaks, and selected candidate SNPs in these regions. Finally, we performed association analyses on the candidate SNPs, using both FBAT and MDR.

### Datasets

We used the simulated data from the country of Aipotu. The Aipotu families were selected when at least two offspring were present who had P1, P2, or P3. We chose disease status for Kofendrerd Personality Disorder (KPD) as the phenotype of interest. In order to get sufficient sample size for MDR analysis, we combined five replicates (REP001-005) of microsatellite marker and SNP data, with 500 nuclear families. We first performed genome-wide linkage analysis using microsatellite markers to identify candidate regions, then we selected SNPs in the candidate regions for the follow-up association analysis. To simulate discordant sib-pair design, we randomly selected 410 discordant sib pairs (820 individuals), with one discordant sib pair from each family. This dataset was then analysed using FBAT and MDR.

### Linkage analysis

We performed multipoint linkage analysis on microsatellite markers using GENEHUNTER-PLUS. This approach is based upon 1-parameter allele sharing model [8], which allows exact calculation of likelihoods and LOD scores. There are 2 forms of the 1-parameter allele-sharing model, a linear model and an exponential model. We applied an exponential model to calculate the LOD score because it has several nice properties when compared to the linear model [8]. The LOD-score function can be used to construct confidence regions for gene location. We used this feature to identify the candidate regions for the follow-up association analysis. The map provided by GAW14 was in recombination fraction (rf) units, we also use rf as the unit of analysis for the results.

### Association analysis

All 29 SNPs were tested for Hardy-Weinberg Equilibrium (HWE) to check the quality of the data. SNPs not in HWE were removed from the analysis.

The FBAT program implements a series of family-based association tests [1]. When testing for association in an area of known linkage with data from multiple sibs in a family or multiple families in a pedigree, the most appropriate test statistic is based upon the empirical variance. Because we used a positional approach to establish candidate regions, our FBAT test statistics for SNPs were computed using the empirical variance.

### Multifactor-dimensionality reduction (MDR)

MDR [2] is a modification of the combinatorial partitioning method (CPM) [3]. It was developed specifically to detect higher-order interactions among polymorphisms that predict dichotomous trait variation, even when the marginal effects are very small [9]. MDR reduces the dimensionality of multilocus information to improve the identification of polymorphism combinations associated with disease risk. The general steps of MDR method are: 1) partition the data into some number of equal parts for a *v*-fold cross-validation (e.g., 10-fold, depending on the sample size); 2) select a set of *n *candidate genetic and/or discrete environmental factors from all factors; 3) represent the *n *factors and their multifactor classes (*m *genotypes/locus means *n*^*m *^classes) in *n*-dimensional space; 4) estimate the ratio (*R*) of the number of affected sibs (*A*) to the number of unaffected sibs (*U*) within each multifactor class. Each multifactor class in *n*-dimensional space is labelled either as "high-risk," if *R *≥ *T *(some threshold), or as "low-risk," if *R *<*T*, thereby reduces the *n*-dimensional model to a uni-dimensional model. For balanced designs, the threshold is usually set to 1:1. 5) All possible combinations of *n *factors are evaluated sequentially for their ability to classify affected and unaffected individuals in the training data. The best *n*-factor model is selected. 6) The independent test data from the *v*-fold cross-validation is used to estimate the prediction error of the best model selected in Step 5. Steps 1–6 are repeated *v *times with the data split into *v *different training and testing sets.

We analyzed the SNP dataset using MDR as described above, with an affected sibs-to-unaffected sibs threshold ratio of 1 and 10-fold cross-validation. We conducted exhaustive search of all possible 1- to 7-locus interactions. Due to the fact that the number of possible combinations is exponential to the number of loci tested for interaction, it is computationally overwhelming to do an exhaustive search of all higher order interactions. Cross-validation consistency (CVC) was used as the statistic to select the best model [9]. Empirical *p*-values of MDR results were obtained by running 100 permutation tests.

## Results

### Linkage analysis

Four linkage peaks were identified in the multipoint linkage analysis on chromosomes 1 (1.62 rf, LOD = 7.25, *p *= 3.73 × 10^-9^), chromosome 3 (2.9 rf, LOD = 14.3, *p *< 10^-14^), chromosome 5 (0.058 rf, LOD = 7.3, *p *= 3.37 × 10^-9^) and chromosome 9 (0.06 rf, LOD = 3.56, *p *= 2.55 × 10^-5^). We used the 1-LOD score rule to get the 90% confidence regions as candidate regions. Because there was no uniform map for microsatellite markers and SNPs, we assumed two extreme scenarios in which either left ends of the two maps align, or the right ends of the two maps align, and then defined the combination of the candidate regions in both situations as the final candidate regions. We then selected the 29 SNPs (Table 1) in these regions for association analysis using FBAT and MDR. Twenty-nine SNPs were each given a number for reference in the following comparison (Table 1).

### Comparison of results from FBAT and MDR

All of the 29 SNP were in HWE (*p *> 0.05), so all of them are included in the analyses. Based on real answers, four susceptibility loci (D1 to D4) were within the candidate regions; two susceptibility loci (D5 and D6) on chromosomes 10 and 2 were not in the candidate regions, hence were not included in the follow-up association analysis. D1, D2, D3, and D4 were within the candidate regions, but do not appear in the map. They are in the extra SNP packets, which we did not order at the time of our analysis. D1, D2, D3, and D4 cause disease via four 2-locus interactions (D1–D2, D2–D3, D3–D4, and D1–D4). The closest SNPs to the four susceptibility loci in our map (6^th^, 16^th^, 19^th^, and 25^th^) were referred to as D1 to D4 for our purposes, unless otherwise noted. Using FBAT, we identified D4 (*p *= 0.043). However, after correcting for multiple tests, it is not significant (*p *= 0.129, false discovery rate (FDR) correction on chromosome 9). Using MDR, we did not identify any of 1- to 7-locus models as significantly different from the null hypothesis of no association. According to cross-validation consistency, the MDR 6-locus model was the best model (*p *= 0.84) including interaction of D1–D2 (Table 2). Compared with the answers, our 7-locus model was the best model. Using this model, we detected the interactions D1–D2 and D2–D3, although only directionally.

## Discussion

We compared FBAT and MDR in their ability to detect susceptibility loci using discordant sib-pair dataset generated from GAW14 simulated data. Using FBAT, our most promising finding was for one of the major loci, D4. Using MDR, our most promising finding was a 7-locus model including D1, D2, and D3. However, none of our results reached a level of statistical significance allowing us to reject the null hypothesis of no association.

Our family-based analysis using FBAT did not result in a statistically significant association with any of the susceptibility loci. This may be because the algorithm is designed to find loci with main effects, not loci interacting with one another, which was the case in the GAW14 simulation dataset. It is also possible that when we randomly selected discordant sib pairs, we lost power we would have had in analyses using the full pedigree information.

There are several possible explanations for the lack of power for MDR in these analyses. Heterogeneity may have played a role. In the simulated data, there were four 2-locus interactions (D1–D4, D1–D2, D2–D3, and D3–D4); each interaction caused disease alone. It has been shown by simulation that MDR has very limited power in the presence of heterogeneity [9], ranging from 5% to 41%, regardless of the particular epistasis model. The "Aipotu" families had P1, P2, or P3, each of which is caused by one of the four interactions. This heterogeneity may explain why we were unable to achieve statistical significance using MDR. Restricting the analyses to clusters of individuals with similar phenotypes prior to analysis by MDR may be one way to overcome the limitation of this method for dealing with genetic heterogeneity. All SNPs were tested for pair-wise linkage disequilibrium (LD). There was none present in the simulated sample. Our results were not influenced by the presence of LD. The best model with most susceptibility loci included (D1, D2, D3). This model had the highest dimensionality (7-locus) of all we tested. It may be that higher-dimensional models may have to be tested in order to include all susceptibility loci. However, the combinatorial nature of MDR makes it impractical to test very high-dimensional models. Other statistical methods may be used to identify a smaller set of candidate loci in order to leverage this benefits this method offers. The final possible explanation for our loss of power is our choice for the sampling scheme and loci examined. Our candidate regions did not include D5 or D6. These affected the penetrance of trait P2, which was caused by interactions between D2–D3 or D3–D4. Our sampling procedure randomly selected discordant sib-pairs. In doing so, we omitted information from other pedigree members.

## Conclusion

MDR has been shown to be useful in the identification of gene × gene interactions in real data from case-control studies [2,6]. In order to examine the efficacy of this method for a discordant sib-pair design we compared FBAT and MDR using the GAW14 simulated data. We found that neither FBAT nor MDR, with 1- to 7-locus models, were able to detect the susceptibility loci in discordant sib-pair dataset. FBAT is designed to find loci with main effects, not interactions, so we expected that we would not detect interacting loci. Our MDR models did not detect the susceptibility loci either, likely due to genetic heterogeneity and our sample design. In most epidemiological studies, the genetic variations conferring liability for disease are unknown, and not all candidate factors can be selected. Methods for dealing with heterogeneity may be successful when homogeneous sub-phenotypes can be defined. The candidate gene approach is common, but there might still be hundreds to thousands of candidate SNPs. Extra steps will likely be necessary to reduce the number of SNPs examined for association. MDR has been shown to be useful in case-control studies when there is no heterogeneity, but has limited power where heterogeneity is present [2,7]. We have shown that the MDR approach does not have the power to detect interactions in the presence of more complex heterogeneity in a discordant sib-pair dataset. However, our directional detection of two interactions (D1–D2, D2–D3) in a 7-locus model may suggest that this approach might be successful in other settings.

## Abbreviations

CPM: combinatorial partitioning method

CVC: Cross-validation consistency

FBAT: Family-based association test

FDR: False discovery rate

GAW14: Genetic Analysis Workshop 14

HWE: Hardy-Weinberg Equilibrium

KPD: Kofendrerd personality disorder

LD: Linkage disequilibrium

MDR: Multifactor-dimensionality reduction

SNP: Single-nucleotide polymorphism

## Authors' contributions

YM participated in the design of the study, performed the statistical analysis and drafted the manuscript. QM, YY, and JF participated in its design and coordination. LAF participated in the design of the study. MAW participated in the design of the study and helped to draft the manuscript. All authors read and approved the final manuscript.

## Figures and Tables

**Table 1:** 29 candidate SNPs identified by linkage peaks.

Chromosome 1		Chromosome 3		Chromosome 5		Chromosome 9	
			
SNP	Number	SNP	Number	SNP	Number	SNP	Number
C01R0047	1	C03R0275	10	C05R0378	17	C09R0763	23
C01R0048	2	C03R0276	11	C05R0379	18	C09R0764	24
C01R0049	3	C03R0277	12	**C05R0380**	**19 (D3)**	**C09R0765**	**25 (D4)**
C01R0050	4	C03R0278	13	C05R0381	20	C09R0766	26
C01R0051	5	C03R0279	14	C05R0382	21	C09R0767	27
**C01R0052**^**a**^	**6 (D1)**	C03R0280	15	C05R0383	22	C09R0768	28
C01R0053	7	**C03R0281**	**16 (D2)**			C09R0769	29
C01R0054	8						
C01R0055	9						

^a^Bold text indicates real susceptibility loci.

**Table 2:** Results of MDR analysis of 29 SNPs.

Number of factors considered	Best candidate model	Susceptibility loci identified	Cross-validation consistency	Mean Classification error %	Mean Prediction error %
1	8	-	5	46.1	53.41
2	5, 12	-	4	43.88	52.01
3	5, 12, 14	-	3	40.51	50.12
4	4, 5, **19**^**a**^, 24	D3	2	35.95	54.14
5	4, 5, **6**, 10, 24	D1	2	28.54	54.10
6	4, 5, **6**, 10, **16**, 24	D1, D2	**6**	19.38	51.52
7	4, **6**, 14, **16**, 18, **19**, 24	D1, D2, D3	3	10.95	50.44

^a^Bold text indicates real susceptibility loci.

## References

[B1] Horvath S, Xu X, Laird N (2001). The family based association test method: strategies for studying general genotype-phenotype associations. Eur J Hum Gen.

[B2] Ritchie MD, Hahn LW, Roodi N, Bailey LR, Dupont WD, Parl FF, Moore JH (2001). Multifactor-dimensionality reduction reveals highorder interactions among estrogen-metabolism genes in sporadic breast cancer. Am J Hum Genet.

[B3] Hoh J, Wille A, Ott J (2001). Trimming, weighting, and grouping SNPs in human case-control association studies. Genome Res.

[B4] Nelson MR, Kardia SL, Ferrell RE, Sing CF (2001). A combinatorial partitioning method to identify multilocus genotypic partitions that predict quantitative trait variation. Genome Res.

[B5] Hoh J, Ott J (2003). Mathematical multi-locus approaches to localizing complex human trait genes. Nat Rev Genet.

[B6] Moore JH, Williams SW (2000). New strategies for identifying gene-gene interactions in hypertension. Ann Med.

[B7] Ritchie MD, Hahn LW, Moore JH (2003). Power of multifactor dimensionality reduction for detecting gene-gene interactions in the presence of genotyping error, phenocopy, and genetic heterogeneity. Genet Epidemiol.

[B8] Kong A, Cox NJ (1997). Allele-sharing models: LOD scores and accurate linkage tests. Am J Hum Genet.

[B9] Hahn LW, Ritchie MD, Moore JH (2003). Multifactor dimensionality reduction software for detecting gene-gene and gene-environment interactions. Bioinformatics.

